# Non-Targeted Hyperspectral Imaging Screening of Adulterants and Congeneric Species in *Fritillaria* Using a Deep Spectral Autoencoder

**DOI:** 10.3390/foods15112014

**Published:** 2026-06-04

**Authors:** Zhizhi Huang, Kai Chen, Haoyuan Ding, Zhangting Wang, Yilei Zhang, Huangwei Li, Ziyuan Liu, Fan Yan, Yujia Dai

**Affiliations:** College of Optical, Mechanical and Electrical Engineering, Zhejiang A&F University, Hangzhou 311300, China

**Keywords:** hyperspectral imaging, *Fritillaria*, adulteration detection, congeneric species, deep autoencoder, anomaly detection

## Abstract

Hyperspectral imaging has emerged as a powerful tool for food quality assessment, yet most existing methods rely on supervised classification and require prior knowledge of adulterant categories. This study applies a non-targeted screening approach based on a deep spectral autoencoder to detect adulterants in *Fritillaria*. While autoencoder-based anomaly detection has been established in other hyperspectral domains, its application to congeneric species discrimination and exogenous adulterant screening in *Fritillaria* has not been systematically explored. A deep spectral autoencoder was constructed and trained exclusively on pure samples to learn the intrinsic spectral distribution of authentic materials. During inference, reconstruction error was used as an anomaly score, and samples deviating from the learned spectral manifold were identified as suspicious. Spectral data augmentation and band trimming were applied to enhance model robustness, while the anomaly threshold was determined solely from the distribution of pure samples. The proposed method achieved strong discrimination performance, with an area under the receiver operating characteristic curve (AUC) of 0.9903 and high detection rates across multiple adulterant types. Typical exogenous adulterants such as starch and talc powder were completely detected, while congeneric species also showed high detection sensitivity despite their spectral similarity to authentic samples. Latent space visualization and residual spectral analysis further revealed clear separation patterns and interpretable spectral deviations. These results demonstrate the proof-of-concept viability of the proposed non-targeted framework for open-set screening of adulteration risks. However, the authentic samples used for training originated from a single source, and only a limited set of anomaly types was tested. Therefore, the current model should be regarded as an early proof-of-concept only, not as a ready-to-deploy screening tool. Further validation with diverse authentic samples and a wider range of adulterants under realistic variability is necessary before the method can be considered a practical strategy for quality control.

## 1. Introduction

Hyperspectral imaging (HSI) has become an increasingly important tool for rapid, non-destructive quality assessment because it simultaneously captures spatial and spectral information and can be combined with chemometrics or machine learning for detecting adulteration, contamination, and non-conformity in complex materials [1,2,3,4,5]. In the context of food and medicinal products, recent reviews have highlighted HSI as a promising platform for quality control and safety inspection [6,7,8,9,10]. Within the broader food-medicine homology framework, recent perspectives and reviews have emphasized the transition from traditional medicine-food homology to modern, science-based, and standardized food-medicine homology, while also highlighting traditional Chinese medicine resources as functional foods [11,12,13].

Against this backdrop, *Fritillaria* is a valuable traditional medicinal material widely used for its antitussive and expectorant properties, but its high economic value and limited supply make it vulnerable to adulteration and substitution [14,15,16]. Recent studies have shown that *Fritillaria* cirrhosae bulbus is frequently adulterated with closely related species, and that commercially cheaper species or powders may be mixed into authentic materials for economic gain [17]. Molecular and nucleic-acid-based authentication studies have therefore been developed to distinguish *Fritillaria* species and their adulterants, underscoring the practical importance of reliable quality control [18,19]. In recent years, HSI combined with artificial intelligence has been explored for *Fritillaria* authentication and quality control. For example, an attention-enhanced one-dimensional convolutional neural network (1D-CNN) model was reported to provide reliable, efficient, and non-destructive identification of *Fritillaria* species [20], while another HSI-based study demonstrated the potential of AI-assisted spectral analysis for variety discrimination of *Fritillaria* thunbergii [21]. These studies support the feasibility of HSI-based authentication, but they mainly focus on distinguishing predefined species or known categories. However, most existing HSI-based authentication frameworks are supervised and rely on prior knowledge of all target classes and sufficient labeled samples for each category [22,23]. In practical quality control scenarios, this assumption is often unrealistic because unknown adulterants, emerging substitutes, and subtle processing-related variations may appear unexpectedly. As a result, supervised classifiers may struggle in open-set conditions, especially for *Fritillaria* materials in which congeneric species can exhibit high spectral similarity to authentic samples. To the best of our knowledge, no study has yet applied autoencoder-based one-class anomaly detection to non-targeted adulterant screening in *Fritillaria*, especially for detecting both exogenous fillers and congeneric species without prior knowledge of adulterant types. The present work therefore provides a case study of how an established anomaly detection framework can be adapted to this specific food–medicine homology quality control context.

To address this limitation, non-targeted screening strategies have attracted growing attention [24,25,26]. Among them, reconstruction-based anomaly detection using autoencoders is particularly appealing because the model can be trained only on normal samples, learn the intrinsic data manifold, and then identify samples with large reconstruction errors as anomalies [27]. Recent studies on hyperspectral anomaly detection have shown that autoencoder-based approaches can be effective under complex and noisy conditions, providing a useful basis for open-set screening tasks [28,29].

**Figure 1:**
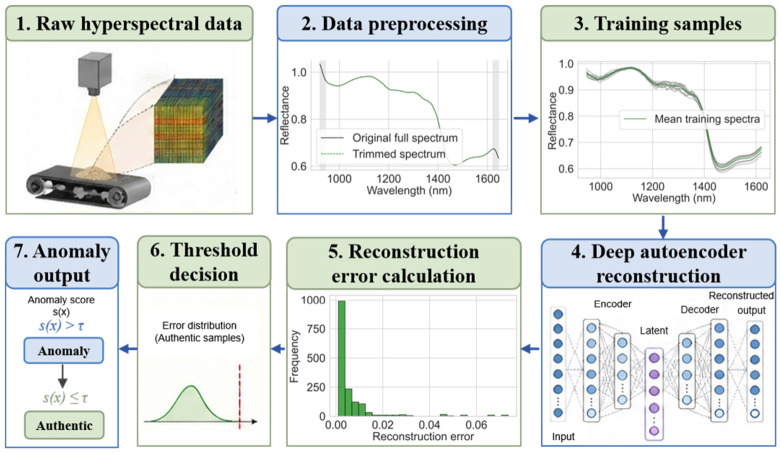
Workflow of the proposed non-targeted hyperspectral screening framework for *Fritillaria*.

Based on this idea, the present study applies a deep spectral autoencoder to *Fritillaria* adulteration screening as a proof-of-concept, as shown in Figure 1. Our contribution is its application and domain-specific adaptation to *Fritillaria* adulteration screening using a limited set of test cases (starch, talc, and four congeneric species). The model is trained exclusively on authentic *Fritillaria* samples to learn their stable spectral characteristics, while reconstruction error is used as an anomaly score to identify adulterated and substituted samples. To improve robustness, spectral preprocessing and data augmentation strategies are incorporated, and the anomaly threshold is determined solely from the distribution of normal samples. The proposed method is evaluated on multiple types of abnormal samples, and latent space visualization together with residual spectral interpretation is used to examine the learned feature structure and the wavelength regions contributing to anomaly detection. We emphasize that the current work does not claim general open-set detection ability; such claims would require substantially broader validation.

## 2. Materials and Methods

### 2.1. Sample Preparation

**Figure 2:**
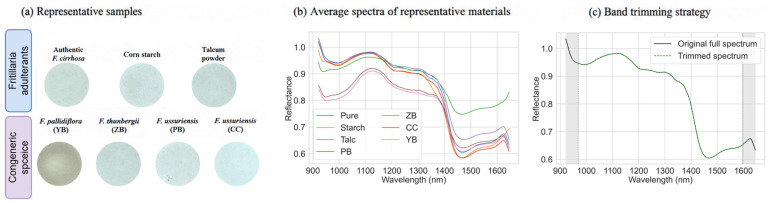
Representative samples and preprocessing strategy for spectral data.

Authentic *Fritillaria cirrhosa* D. Don bulbs were purchased from a local pharmacy and used as the reference authentic material. As shown in Figure 2a, corn starch and talcum powder, obtained from certified suppliers, were selected as representative foreign adulterants because they are commonly used as low-cost fillers and exhibit spectral characteristics that differ markedly from those of authentic *Fritillaria*. In addition, four congeneric materials were included to evaluate the ability of the proposed model to distinguish closely related species: *Fritillaria pallidiflora* Schrenk (Yibei, YB), *Fritillaria thunbergii* Miq. (Zhebei, ZB), and two commercial varieties of *Fritillaria ussuriensis* Maxim., namely Pingbei (PB) and Changchunbei (CC). Although PB and CC share the same botanical origin, they were treated as separate classes to examine whether the proposed method is sensitive to differences arising from geographical origin or commercial grading.

Before spectral acquisition, all samples were dried at 60 °C and then pulverized for 5 min using a high-speed grinder (Anka, Zhongshan, China). The resulting powders were used directly without sieving in order to preserve the particle-size heterogeneity typically encountered in practical market samples. For the foreign-matter adulteration set, binary mixtures were prepared by blending pure *F. cirrhosa* powder with starch or talc at mass fractions ranging from 0% to 100% with a step size of 10%. Each mixture had a total mass of 5 g and was thoroughly homogenized before measurement. For the congeneric adulteration set, pure *F. cirrhosa* powder was mixed with each congeneric species at mass fractions of 25%, 50%, and 75%, with the total mass of each mixture also fixed at 5 g. All prepared powder samples were stored in sealed polyethylene bags under dry and dark conditions until hyperspectral data acquisition.

### 2.2. Hyperspectral Imaging Acquisition and Spectral Extraction

Hyperspectral images were acquired using a near-infrared hyperspectral imaging system (GaiaField-N17 E, Dualix, Wuxi, China) [30]. The system consisted of a hyperspectral camera covering the spectral range of 900–1700 nm, four 50 W halogen lamps arranged at 60° to provide uniform illumination, an enclosed imaging chamber (HSIA-BD), and a computer workstation equipped with SpecView V1.0 software for data collection and control. The camera provided a spectral resolution of 5 nm, corresponding to 512 spectral bands, and a spatial resolution of 640 pixels.

During acquisition, the powder samples were evenly spread on a conveyor belt with a black background. The scanning parameters were set as follows: moving speed, 6 mm/s; scanning length, 150 mm; exposure time, 7 ms; and lens-to-sample distance, 15 cm. To reduce the influence of dark current and illumination non-uniformity, raw hyperspectral images were corrected using the standard reflectance calibration procedure with a white reference obtained from a 99% Teflon board and a dark reference collected by covering the lens. The calibrated reflectance image was calculated as [31]:

(1) $$I_{corr}=\frac{I_{raw}-I_{black}}{I_{white}-I_{black}}$$

where *I_corr_* denotes the corrected reflectance image, *I_raw_* is the raw image, *I_white_* is the white reference image, and *I_black_* is the dark reference image.

After calibration, regions of interest (ROIs) were extracted from the central area of each sample image using ENVI 5.3 software to avoid edge effects and uneven illumination [32]. For each physical powder sample, 40 independent ROIs were randomly selected, and the mean spectrum of all pixels within each ROI was calculated. Importantly, the 40 spectra obtained from the same physical sample were averaged to produce a single representative spectral signature for that sample. The training and testing sets were then partitioned strictly at the physical sample level; i.e., all representative spectra originating from one physical sample were assigned either entirely to the training set or entirely to the testing set. This ensures that no spectral information from the same physical sample appears in both training and testing, thereby avoiding data leakage and pseudoreplication. The average spectra of representative materials are shown in Figure 2b. A total of 33 physical powder samples were prepared: 21 samples for the foreign-adulterant set (starch and talc mixtures at 10% increments, plus one pure *F. cirrhosa* sample) and 12 samples for the congeneric adulterant set (four species × three mixing ratios). For each physical sample, 40 ROIs were extracted, yielding 1320 ROI-level spectra (33 × 40). These 1320 ROI spectra were then averaged per physical sample to obtain 33 representative spectra, each serving as the independent experimental unit for subsequent model training and validation. The extracted spectra were then arranged into tabular form and subsequently divided into training and testing sets for model development.

### 2.3. Data Preprocessing

To ensure stable model training and reliable anomaly discrimination, the extracted spectral data were further preprocessed in a unified manner. First, the spectral measurements and their corresponding labels were arranged into a tabular form, and the samples were categorized into two groups: authentic and abnormal. Authentic samples were used only for model training, whereas abnormal samples were reserved for testing and performance evaluation.

As shown in Figure 2c, because the two ends of hyperspectral spectra are often more affected by sensor noise, illumination fluctuation, and boundary effects, the first and last 15 bands were removed from the original spectra [33]. This operation reduced the influence of low-quality spectral regions and emphasized the more stable bands associated with chemical composition. After trimming, the remaining spectra were used as the input features of the model. Standardization was applied band-wise using training set statistics:

(2) $$x_{ij}^{\prime }=\frac{x_{ij}-\mu_{j}}{\sigma_{j}}$$

where $\mu_{j}$ and $\sigma_{j}$ are the mean and standard deviation of the training set at that band j. This removes scale differences and emphasizes spectral shape. Since the present study followed a one-class learning strategy, a mild data augmentation procedure was applied only to the authentic training spectra. Specifically, small Gaussian noise was added to the standardized authentic spectra to generate augmented samples. This strategy broadens the local distribution of normal samples, improves robustness to measurement noise and sample heterogeneity, and reduces the risk of overfitting to incidental variations [34]. The final preprocessed data were then divided into training and testing sets, with the split performed at the physical-sample level. The training set contained only authentic physical samples, and the testing set included authentic, known adulterant, and congeneric samples for open-set anomaly evaluation.

### 2.4. Deep Autoencoder Model and Anomaly Determination

**Figure 3:**
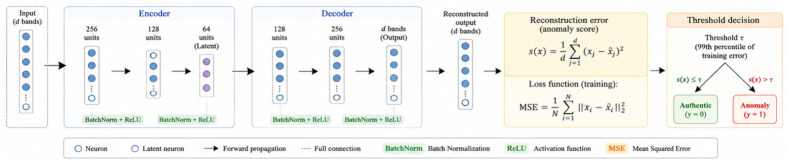
Architecture of the deep spectral autoencoder for anomaly detection.

To realize non-targeted anomaly screening of *Fritillaria* samples, a deep autoencoder-based hyperspectral reconstruction model was developed as shown in Figure 3. Unlike conventional supervised classification methods, the proposed model was trained exclusively on authentic samples so that it could learn the intrinsic spectral manifold of normal *Fritillaria*. At inference time, samples that deviated substantially from this learned manifold were expected to yield larger reconstruction errors and could therefore be identified as potential anomalies. Let $x\in R^{d},$ denote an input spectral vector, where *d* is the number of retained bands. The autoencoder consists of an encoder $f_{\theta }$ mapping x to a latent representation z, and a decoder $g_{\phi }$ reconstructing $\hat{x}=g_{\phi }(f_{\theta }(x))$. The network was trained exclusively on authentic samples by minimizing the MSE reconstruction loss:

(3) $$L_{rec}=\frac{1}{N}\sum_{i=1}^{N}\|x_{i}-{\hat{x}}_{i}{\|}_{2}^{2}$$

Because the model only observes authentic spectra, it learns their intrinsic spectral manifold; adulterated or substitute samples are expected to yield larger reconstruction errors. The anomaly score for an individual sample was defined as the mean squared reconstruction error:

(4) $$s(x)=\frac{1}{d}\sum_{j=1}^{d}(x_{j}-{\hat{x}}_{j}{)}^{2}$$

A sample was classified as abnormal when its anomaly score exceeded a predefined threshold τ:

(5) $$\hat{y}=\left\{\begin{array}{c} 1,s(x)>\tau \\ 0,s(x)\leq \tau \end{array}\right.$$

where $\hat{y}=1$ indicates an abnormal sample and $\hat{y}=0$ indicates an authentic sample. The threshold τ was determined solely from the reconstruction error distribution of authentic training samples, using a high percentile value, thereby avoiding any dependence on labeled abnormal samples and illustrating the potential of the method for deployment pending further validation. This setting is consistent with real-world quality control scenarios, where only normal samples are reliably available and unknown anomalies may emerge unexpectedly.

### 2.5. Evaluation Metrics

To comprehensively evaluate the proposed framework, several metrics were adopted from the perspectives of overall discrimination, class-wise detection, and interpretability. Since this study is a typical anomaly detection task and the testing set may be imbalanced, accuracy alone is insufficient to characterize model performance. Therefore, the receiver operating characteristic (ROC) curves, the area under the ROC curve (AUC) [35], confusion-matrix-based accuracy, and class-specific detection rates were jointly used for evaluation. For the continuous anomaly score s(x), the ROC curve was constructed by plotting the true positive rate (TPR) against the false positive rate (FPR):

(6) $$TPR=\frac{TP}{TP+FN},$$

(7) $$FPR=\frac{FP}{FP+TN}$$

where *TP*, *FP*, *TN*, and *FN* denote true positives, false positives, true negatives, and false negatives, respectively. The AUC was used as a quantitative measure of the model’s overall ability to separate authentic and abnormal samples. An AUC close to 1 indicates strong separability, whereas a value of 0.5 corresponds to random discrimination. After the anomaly threshold was applied, the confusion matrix was computed and the overall accuracy (ACC) was obtained as:

(8) $$ACC=\frac{TP+TN}{TP+TN+FP+FN}$$

Although accuracy provides an intuitive summary of the classification result, it may be influenced by class imbalance. Therefore, it was interpreted together with AUC to provide a more robust assessment of model performance. In addition, the detection rate of each abnormal category was calculated as:

(9) $$DetectionRate=\frac{N_{detected}}{N_{total}}\times 100\%$$

where $N_{detected}$ is the number of abnormal samples correctly identified as anomalies and $N_{total}$ is the total number of samples in that category. This metric is particularly useful for assessing the sensitivity of the model to different adulterants and congeneric species. Finally, to aid interpretability, t-distributed stochastic neighbor embedding (t-SNE) visualization of the latent features and residual spectral analysis were performed [36]. We emphasize that t-SNE is an exploratory visualization tool with inherent sensitivity to parameter choices and stochasticity; therefore, it provides illustrative support rather than rigorous validation. Similarly, residual spectral analysis offers band-level observations that can inform hypotheses about spectral deviations, but definitive assignment to specific chemical or structural causes would require complementary analytical techniques.

### 2.6. Experimental Settings

All experiments were conducted with a fixed random seed to ensure reproducibility. The model was trained using the adaptive moment estimation (Adam) optimizer with an initial learning rate of 1 × 10^−3^. The reconstruction loss was defined as mean squared error (MSE) [37]. The batch size was set to 32, and the model was trained for 200 epochs. During training, the average loss of each epoch was recorded to monitor convergence. The autoencoder adopted a fully connected, gradually compressive architecture. The input spectra were mapped to a latent representation with a dimensionality of 64, which was chosen to balance representation capacity and reconstruction fidelity. After training, the anomaly threshold was determined from the reconstruction error distribution of authentic training samples, with the threshold percentile set to 99%. A comprehensive summary of the network architecture, activation functions, regularization methods, and training settings is provided in Table 1.

During testing, the spectra were fed into the trained autoencoder to compute sample-wise reconstruction errors, which were then compared with the threshold to obtain anomaly predictions. ROC curves and AUC values were calculated from the anomaly scores, and the detection rates of different categories were summarized. For representative abnormal samples, residual spectra were obtained by subtracting the reconstructed spectra from the original spectra, enabling band-level interpretation of the anomaly mechanism. In addition, the latent feature space was projected into two dimensions using t-SNE to visualize the distribution differences between authentic and abnormal samples.

## 3. Results

To evaluate the effectiveness of the proposed deep spectral autoencoder for non-targeted anomaly screening of *Fritillaria*, the experimental results were systematically analyzed from multiple perspectives, including model convergence, anomaly discrimination capability, latent feature distribution, and residual spectral response. Unlike conventional supervised classification methods, the core objective of this study is not to finely discriminate predefined categories, but rather to learn the stable spectral distribution of authentic *Fritillaria* and achieve open-set recognition of unknown adulterants, substitutes, and congeneric species. Therefore, the focus of the results analysis is not only on whether the model can correctly distinguish authentic and anomalous samples, but also on its stability in detecting unseen categories and the spectral interpretability of its predictions. Following this rationale, we first analyzed the model’s training loss and ROC/AUC performance to verify its convergence characteristics and overall discriminative ability, and then further examined anomaly detection across different types of abnormal samples, latent space distribution, and residual spectral features to elucidate the underlying recognition mechanisms and practical implications.

### 3.1. Training Loss and ROC Analysis

**Figure 4:**
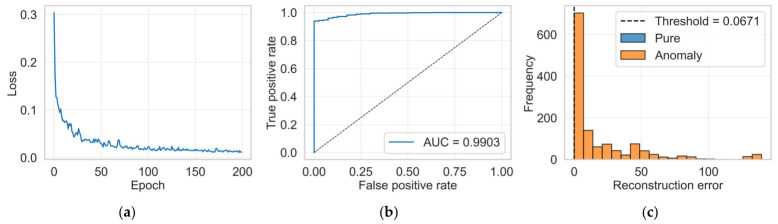
Training convergence and overall anomaly discrimination performance of the proposed deep spectral autoencoder: (**a**) training loss curve, (**b**) ROC curve, and (**c**) reconstruction error histogram with the anomaly threshold line.

As shown in Figure 4a, the training loss decreased rapidly and stabilized, indicating convergence of the autoencoder on the authentic spectral manifold. Since the model essentially approximates the normal spectral manifold of authentic samples through a “compress–reconstruct” mechanism, the persistent decrease in the loss function demonstrates the model’s improving ability to represent the normal spectral distribution and gradually forms a stable reconstruction mapping.

Regarding anomaly discrimination, the ROC curve plotted based on reconstruction errors of test samples (Figure 4b) is significantly above the random baseline and approaches the top-left corner, indicating strong discriminative capability between authentic and anomalous samples. The corresponding AUC reaches 0.9903, demonstrating that reconstruction error serves as a highly effective anomaly score and that the method maintains robust performance across different threshold settings. Unlike conventional supervised classification methods, this reconstruction-error-based anomaly detection framework does not rely on prior labels of anomalies, making it well-suited for open-set, non-targeted screening tasks.

**Figure 5:**
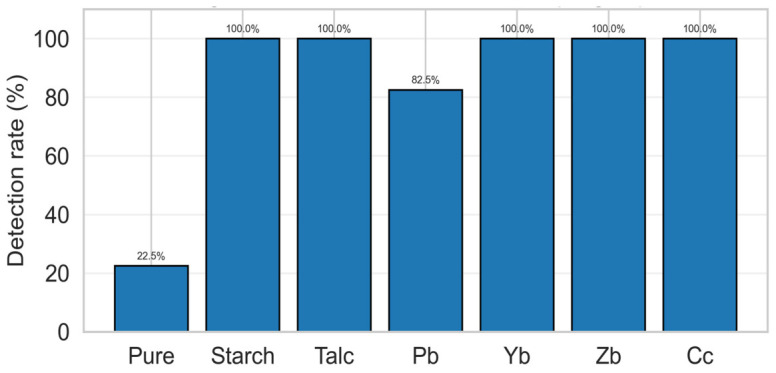
Detection rates of different sample groups under the proposed non-targeted hyperspectral screening framework, including authentic samples, starch, talc, Pb, Yb, Zb, and Cc.

Examining the distribution of reconstruction errors (Figure 4c), authentic and anomalous samples show clear separation. Reconstruction errors for authentic samples are concentrated in a low-value region, whereas anomalous samples shift toward higher values with extended tails, reflecting significant deviations from the learned normal spectral manifold. A 99th percentile threshold of 0.0671, determined from the training set, effectively separates most authentic and anomalous samples. It should be noted that although this threshold is highly sensitive to anomalies, it introduces a small number of false positives among authentic samples, which is more clearly illustrated in Figure 5. Overall, the stable convergence of training loss, the excellent ROC performance, and the clear separation of reconstruction error distributions collectively demonstrate that the proposed deep autoencoder can reliably identify anomalies in non-targeted screening tasks and provide a foundation for further stratified analysis of different anomaly types.

### 3.2. Detection of Different Anomaly Types

To further evaluate the model’s ability to detect different types of anomalies, the anomaly detection rates were calculated for each category and compared. Figure 5 presents the detection results for authentic samples, starch, talc, and congeneric species. Compared with the overall AUC metric, category-specific detection results more directly reflect the model’s sensitivity to different anomaly patterns, particularly its ability to consistently detect exogenous fillers with marked chemical differences versus congeneric species with more similar spectral features. The results indicate that the model is highly sensitive to typical low-cost adulterants. Both starch and talc achieved 100% detection rates, demonstrating that these exogenous fillers exhibit significant spectral deviation from authentic *Fritillaria*, leading to large reconstruction errors and reliable anomaly identification. This shows that the autoencoder has effectively learned the stable spectral constraints of authentic *Fritillaria* and can robustly screen samples deviating from this constraint. For congeneric species, the model also achieved strong detection performance. PB reached 82.5% detection, while YB, ZB, and CC all achieved 100%. Despite the high spectral similarity of congeneric species to authentic samples, systematic differences in chemical composition, tissue structure, or moisture content still exist, resulting in distinguishable reconstruction errors. Compared with starch and talc, these spectral deviations are subtler, making detection more challenging; the slightly lower detection rate of PB indicates that it is a more difficult anomaly type in this study. Nevertheless, the model maintains high overall recognition performance, indicating that it can detect both prominent adulteration and subtle, persistent spectral shifts. Notably, 22.5% of authentic samples were misclassified as anomalies; i.e., 9 out of 40 authentic samples exceeded the threshold. This false positive rate (FPR) is substantially higher than what would be acceptable in any practical quality control workflow, where repeatedly flagging one in four genuine products would lead to unnecessary re-testing, economic loss, and loss of operator trust. While the current threshold (99th percentile of training reconstruction errors) was intentionally set to prioritize sensitivity over specificity, the resulting FPR of 22.5% indicates that this trade-off is far from optimal for real-world deployment. It should also be noted that this elevated false positive rate may reflect the narrow distribution of authentic training samples (single source), rather than being solely a threshold selection issue. Increasing the diversity of authentic samples in future studies could help reduce this rate while maintaining sensitivity. Several factors likely contribute to this high FPR. First, the authentic training samples originated from a single commercial batch, so the model may have learned batch-specific spectral idiosyncrasies rather than the full range of natural variation. Second, the threshold was derived solely from the training distribution without any calibration using independent authentic samples. Third, the data augmentation strategy (adding Gaussian noise) may not adequately simulate real sources of variability such as differences in moisture, particle size, or measurement conditions. Therefore, while the current threshold setting is conceptually defensible for a proof-of-concept, it is not suitable for practical deployment. In practical terms, flagging nearly one in four authentic samples would generate an unacceptable number of unnecessary confirmatory tests and erode operator confidence. Consequently, the proposed method must be viewed as an early proof-of-concept demonstration, not as a production-ready system. Future work must focus on reducing the FPR through more representative authentic data, adaptive thresholding, or alternative anomaly scoring mechanisms before any practical deployment can be considered. The following sections discuss these issues in greater depth.

In summary, Figure 5 demonstrates that the proposed deep autoencoder, trained on authentic sample distributions, achieves near-perfect detection of low-cost fillers while retaining strong screening capability for congeneric species. These results further validate the effectiveness of reconstruction error as an anomaly score and highlight the potential of this approach for open-set screening of traditional medicinal herbs such as *Fritillaria*.

### 3.3. t-SNE Latent Space Analysis

**Figure 6:**
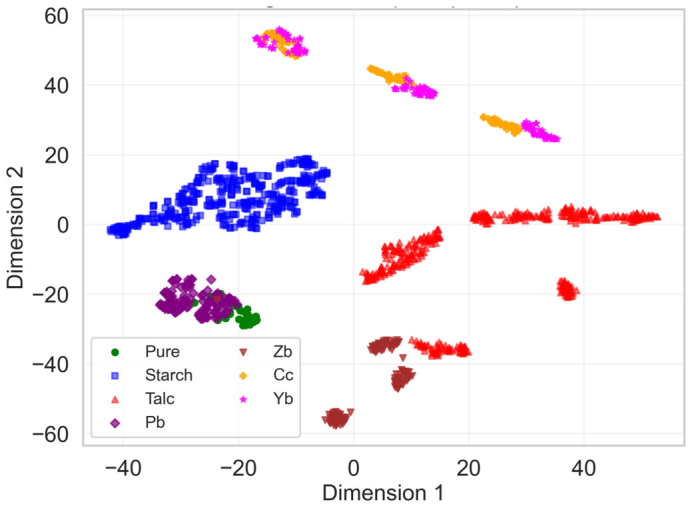
t-SNE visualization of latent features extracted by the deep spectral autoencoder, showing the distribution of authentic samples and different abnormal sample groups in the learned feature space.

To provide an illustrative visualization of the encoder outputs, t-SNE was applied to project the latent representations into two dimensions. It should be noted that t-SNE is an exploratory visualization tool; its results depend on hyperparameters (e.g., perplexity) and stochastic optimization, and it does not provide quantitative validation of feature learning. With this caveat in mind, Figure 6 shows that in this particular embedding, authentic samples appear to cluster in a central region, while many anomalous samples are positioned further away. This visualization is consistent with the reconstruction-error-based anomaly detection results, but it should not be interpreted as rigorous evidence that the encoder has learned ‘stable’ or ‘meaningful’ representations in an absolute sense. The observed separation is sensitive to t-SNE parameters and may not be fully reproducible under different settings.

From the perspective of different anomaly types, low-cost fillers such as starch and talc are typically far from the cluster center of authentic samples and form clear boundaries in 2D space, reflecting substantial chemical and spectral deviations. Congeneric species such as PB, ZB, CC, and YB, on the other hand, have latent representations closer to authentic samples, with some overlap. This aligns with expectations, as congeneric species are more spectrally similar, requiring the model to detect subtle spectral differences. Nevertheless, these samples still tend to deviate from the core region of authentic samples, indicating that the autoencoder captures fine-grained spectral deviations while learning the normal spectral manifold. The t-SNE visualization further validates the method’s effectiveness. The separation between authentic and anomalous samples in latent space confirms that the encoder has learned meaningful low-dimensional representations rather than merely compressing noise. The distribution differences among anomaly types reveal not only binary anomaly detection but also the heterogeneity of anomaly sources. Local overlaps and transitional boundaries in latent space also highlight that anomaly detection is based on continuous deviation from normal distributions rather than absolute segmentation, consistent with the reconstruction-error-based scoring mechanism.

### 3.4. Residual Spectral Analysis

**Figure 7:**
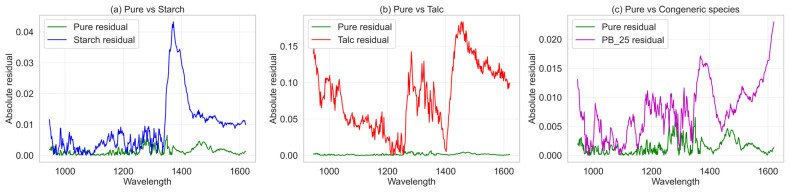
Residual spectral profiles of authentic and representative abnormal samples: (**a**) pure sample versus starch, (**b**) pure sample versus talc, and (**c**) pure sample versus congeneric species.

To further investigate the spectral features relied upon for anomaly detection, residual spectra of authentic and representative anomalous samples were analyzed. The residual spectrum, defined as the absolute difference between original and reconstructed spectra, reflects the extent to which a sample deviates from the learned normal spectral pattern. Compared with overall reconstruction errors, residual spectra provide finer, band-level interpretability, highlighting key regions contributing to anomaly detection. As shown in Figure 7, residuals for authentic samples are generally low and vary smoothly across wavelengths, suggesting that the autoencoder reconstructs the main spectral structure of authentic *Fritillaria* relatively well. In contrast, residuals for anomalous samples are substantially higher, with prominent peaks at specific wavelengths, highlighting spectral regions where deviations from the learned normal manifold are most pronounced. In the NIR region (900–1700 nm), absorption bands are primarily associated with overtones and combinations of fundamental vibrations of O–H, C–H, N–H, and S–H groups. For starch (Figure 7a), elevated residuals are observed around 980 nm (O–H second overtone), 1200 nm (C–H second overtone), and 1450 nm (O–H first overtone), which are characteristic of carbohydrate structures [38]. Talc (Figure 7b) shows strong residuals near 1390 nm (O–H combination band) and 1400–1500 nm (hydroxyl stretching overtones), consistent with its magnesium silicate hydrate composition. For congeneric species (Figure 7c), residual peaks are less pronounced but still detectable around 1200 nm and 1450 nm, suggesting subtle differences in C–H and O–H environments between authentic *Fritillaria cirrhosa* and other *Fritillaria* species. These differences may arise from variations in the relative concentrations of alkaloids, saponins, and polysaccharides among species.

The residual patterns differ across anomaly types. Low-cost fillers such as starch and talc exhibit elevated residuals across multiple bands. Congeneric species also display higher residuals compared with authentic samples, but peaks are more concentrated in certain spectral regions. These observations are consistent with the expectation that exogenous fillers have larger spectral differences from authentic *Fritillaria* than congeneric species do. However, we caution that without independent chemical or physicochemical analysis (e.g., reference measurements of specific compounds), any attribution of residual peaks to particular chemical components (such as absorption features or scattering behavior) remains speculative. The residual analysis presented here is intended to provide a qualitative, band-level view of where reconstruction errors occur, not to establish causal chemical interpretations. Future work integrating hyperspectral imaging with targeted chemical analysis could help validate such assignments. Deviations in these regions for anomalous samples lead to elevated reconstruction errors and trigger anomaly detection.

Overall, residual spectral analysis further confirms the interpretability and effectiveness of the proposed method. Low residuals for authentic samples indicate accurate modeling of normal spectra, while significant residuals for anomalies reveal distinguishable spectral deviations. Combined with ROC, AUC, t-SNE, and category-specific detection results, the deep autoencoder provides an interpretable and reliable non-targeted screening framework for quality and safety assessment of *Fritillaria*.

## 4. Discussion

### 4.1. Advantages of Anomaly Screening Based on Normal Distribution Modeling

Unlike conventional multi-class classification, the proposed framework learns the normal spectral distribution of authentic *Fritillaria* and flags deviations. This transforms the problem into a ‘does the sample deviate from normal?’ detection task, which is well suited to open-set scenarios where adulterant types are not exhaustively known. For high-value traditional medicinal herbs like *Fritillaria*, the market may contain low-cost fillers, congeneric substitutes, and novel contamination patterns. Reliance on fixed-category supervised classification often fails to cover all potential risk sources, whereas anomaly detection based on normal distribution modeling can maintain robust screening capabilities without prior knowledge of anomaly labels [39,40,41].

Experimental results indicate that the deep autoencoder can stably learn the dominant spectral structure of authentic *Fritillaria* and generate significantly higher reconstruction errors for anomalous samples during testing. This demonstrates that the model successfully approximates the spectral manifold of authentic samples, and any sample that substantially deviates from this manifold produces noticeable residuals during reconstruction. Unlike classification models that rely on explicit category boundaries, reconstruction-based anomaly detection focuses directly on whether a sample conforms to normal patterns, providing superior generalization potential for previously unseen anomaly types. This is conceptually relevant in regulatory applications, where anomalies often appear as ‘unknown deviations’ rather than predefined categories, highlighting the potential adaptability of the proposed method. Nevertheless, the current work serves only as a proof-of-concept; regulatory deployment would require extensive validation with a far broader range of anomalies and real-world sample variability. Moreover, the anomaly threshold is determined solely from the distribution of reconstruction errors of authentic samples, avoiding the influence of anomalous samples on threshold fitting and thus aligning the method more closely with real-world deployment logic. This setting not only enhances the objectivity of the model but also, in principle, increases its potential utility under conditions of incomplete sample labeling; utility in realistic scenarios will depend on future validation with diverse authentic sample sets. Overall, anomaly screening based on normal distribution modeling offers a robust, flexible, and scalable technical approach for *Fritillaria* quality and safety assessment.

### 4.2. Comparison with Supervised Classification and Method Applicability

Compared with conventional supervised classification methods, the proposed deep autoencoder framework exhibits fundamental differences in problem formulation. Supervised models typically require sufficient samples of both authentic and anomalous categories, with pre-defined labels used for training. While such models can achieve high accuracy under controlled laboratory conditions, their effectiveness strongly depends on the completeness of anomaly categories in the training data. When novel adulterants, substitutes, or samples with spectral deviations not represented in training appear during testing, supervised classifiers may misclassify these samples or forcibly assign them to the closest known category, reducing their effectiveness in risk warning. For *Fritillaria* in actual circulation, such limitations are pronounced because adulteration methods are not fixed, and sample variations in origin, processing, and moisture content can be substantial. In contrast, the proposed method does not attempt to assign anomalies to specific categories. Instead, it prioritizes identifying whether a sample deviates from the normal distribution of authentic samples. This “detect anomalies first, then trace the source” strategy better aligns with the practical needs of quality supervision. For congeneric species, although some local spectral regions may resemble authentic samples, any systematic deviation in the overall spectral pattern can still be detected by the model. Experimental results show that the model exhibits high anomaly sensitivity to low-cost fillers and maintains strong detection for spectrally similar congeneric species, indicating that it captures fine-grained spectral structural deviations rather than relying solely on obvious amplitude differences. Recently, deep autoencoder architectures have been successfully applied to denoising chemical exchange saturation transfer (CEST) spectra, as demonstrated by Kurmi et al. [41], who used a denoising convolutional autoencoder to enhance SNR in CEST imaging. While their work focuses on signal denoising rather than anomaly detection, the underlying principle of learning a low-dimensional manifold of normal spectral data is conceptually similar to our approach. The proposed autoencoder outperforms PCA-based anomaly detection, One-Class SVM, and Isolation Forest in terms of AUC and detection rate, and provides open-set screening capability that supervised classifiers lack. These results underscore the value of reconstruction-based deep learning for non-targeted quality control applications.

Regarding applicability, this approach is conceptually suitable for certain scenarios, but none of these potential applications are currently supported by our data: (1) open-set screening where anomaly categories are difficult to exhaustively enumerate, making anomaly detection more reliable than multi-class classification; (2) cases with limited samples and scarce anomaly labels, where one-class learning significantly reduces data collection cost; and (3) scenarios requiring rapid analysis, where reconstruction error provides a simple, interpretable anomaly score. Therefore, the current work should be viewed as an early proof-of-concept that illustrates the potential of the approach, not as evidence of readiness for any of these scenarios. While supervised classification may achieve finer discrimination among known categories, the proposed method appears more conceptually suited for ‘unknown anomaly defense’ only after extensive further development. At present, the experimental design includes only a limited set of anomaly types; consequently, no claims can be made about its ability to detect truly unknown or emerging adulterants in real-world settings. Broad external validation using independent batches, different suppliers, varied storage conditions, different particle sizes, and a much wider range of adulterants is an absolute prerequisite.

### 4.3. Limitations and Future Work

Despite the promising performance and interpretability demonstrated in the present study, several limitations should be acknowledged, which also indicate directions for future research.

First and most critically, the authentic training samples were obtained from a single local pharmacy. For a one-class anomaly detection framework, the diversity of the normal class is the foundation of the entire approach. Real-world authentic *Fritillaria* varies in geographical origin, harvest year, drying and storage conditions, moisture content, particle size, and supplier. By training on only one batch, the autoencoder likely learned batch-specific spectral characteristics rather than the general features of authentic *Fritillaria*. This narrow diversity is a primary cause of the observed high false positive rate (22.5%), because even authentic samples with normal but slightly different spectral profiles fall outside the learned manifold. Consequently, the model in its current state is not suitable for any practical deployment. Expanding the authentic training set to include multiple origins, processing conditions, and harvest years is an absolute prerequisite for moving beyond proof-of-concept. Until such data are collected and the model is re-evaluated, no claims of practical applicability can be made. Second, the anomaly types evaluated in this study were limited to two exogenous fillers (starch and talc) and four congeneric species. While these represent common adulteration risks for *Fritillaria*, they do not exhaust the potential range of anomalies that may appear in real markets, such as other low-cost plant materials, synthetic compounds, or processed by-products. Consequently, the demonstrated performance should not be overinterpreted as evidence of generalizable ‘unknown anomaly defense.’ Future work must systematically test the framework against a much wider and more diverse set of anomaly types, including those with subtle spectral differences and those not represented in the current study, to establish true open-set screening capability. Third, the residual spectral analysis presented in Section 3.4 is descriptive and exploratory. While it highlights wavelength regions where reconstruction errors are large, we have not performed independent compositional analyses to link these residuals to specific chemical or structural features. Therefore, any interpretation in terms of absorption bands, scattering, or chemical composition should be regarded as hypothesis-generating rather than conclusive. Future studies should combine hyperspectral reconstruction with reference analytical methods (e.g., HPLC, FTIR, or GC-MS) to establish mechanistically grounded interpretations. Finally, beyond methodological improvements, the significance of this work should also be considered within the broader context of food–medicine homology research. Recent studies indicate that this field is evolving from experience-based knowledge toward a more standardized, precise, and technology-driven paradigm [42,43,44]. In this transition, integrating advanced sensing techniques such as hyperspectral imaging with data-driven modeling provides a promising pathway for objective quality evaluation. From this perspective, the proposed non-targeted anomaly detection framework not only serves as a practical tool for *Fritillaria* quality control, but also illustrates a scalable strategy for handling unknown risks in complex food–medicine systems. With further refinement, the integration of anomaly detection performance, spectral interpretability, and compositional mechanisms may contribute to establishing a more general analytical framework for food–medicine homology, supporting its ongoing modernization and scientific development.

The high false positive rate (22.5%) observed in this study is a major barrier to practical application. In real quality control workflows, such a rate would require re-testing or manual inspection of nearly one in four authentic samples, incurring significant time and cost. We recognize that the current manuscript did not critically analyze this issue in sufficient depth. Here we elaborate on the likely causes and potential remedies. The high FPR likely stems from three interrelated factors: (i) limited diversity of authentic training samples—the model was trained on a single batch from one pharmacy, making it sensitive to minor spectral variations that are normal in authentic materials; (ii) threshold selection—the 99th percentile was chosen without explicit optimization for an acceptable FPR; (iii) lack of authentic validation set for threshold tuning—in a pure one-class setting, we deliberately avoided using any authentic testing samples to set the threshold, but this can lead to suboptimal trade-offs. To reduce the FPR while maintaining high sensitivity, future work should: (a) collect authentic samples from multiple origins, harvest years, and processing conditions to expand the normal manifold; (b) employ cross-validation or a hold-out set of authentic samples to calibrate the anomaly threshold (e.g., targeting 95% specificity); and (c) explore probabilistic anomaly scores or ensemble methods to improve robustness. Until such improvements are made, the proposed framework should be regarded as a proof-of-concept rather than a production-ready screening tool.

## 5. Conclusions

This study developed a non-targeted hyperspectral screening framework for *Fritillaria* based on a deep spectral autoencoder, with the aim of detecting both known and unknown adulteration risks under open-set conditions. By training the model exclusively on authentic samples, the intrinsic spectral distribution of genuine *Fritillaria* was effectively learned, and abnormal samples were identified using reconstruction error without requiring prior knowledge of adulterant categories. The proposed approach achieved strong overall discrimination performance, with an AUC of 0.9903, and showed excellent sensitivity to typical low-cost adulterants such as starch and talc, both of which were detected at 100%. The model also retained robust performance for congeneric species with high spectral similarity to authentic samples, including PB, YB, ZB, and CC, demonstrating its ability to screen subtle and diverse anomaly patterns. In addition, t-SNE visualization revealed clear separation between authentic and abnormal samples in the latent space, while residual spectral analysis provided band-level interpretability and highlighted the spectral regions contributing to anomaly detection. Overall, the results indicate that the proposed deep reconstruction-based framework is an effective and interpretable early proof-of-concept tool for non-targeted *Fritillaria* screening. However, a critical limitation is that the authentic training samples originated from a single commercial source, and the false positive rate is unacceptably high for any practical quality control workflow. This narrow diversity and high FPR mean that the model in its current form is not suitable for deployment in real-world settings. The method should therefore be regarded as a proof-of-concept demonstration that requires substantial further work—including collection of diverse authentic samples, threshold optimization, and validation on independent market batches—before any practical application can be considered. Within its current scope, the method offers a promising technical basis for future research.

## Figures and Tables

**Table 1:** Detailed architecture and training settings of the deep spectral autoencoder.

Component		Specification
Input layer		Number of bands after trimming: d = 512 − 2 × 15 = 482
Encoder	Layer 1	Linear(482 → 256) + BatchNorm1d(256) + ReLU
	Layer 2	Linear(256 → 128) + BatchNorm1d(128) + ReLU
	Layer 3 (latent)	Linear(128 → 64) + ReLU
Decoder	Layer 1	Linear(64 → 128) + BatchNorm1d(128) + ReLU
	Layer 2	Linear(128 → 256) + BatchNorm1d(256) + ReLU
	Layer 3 (output)	Linear(256 → 482)
Activation functions		ReLU for all hidden layers; linear activation for output layer
Regularization		BatchNorm1d after each linear layer; no dropout or weight decay
Loss function		Mean squared error
Optimizer		Adam (adaptive moment estimation)
Learning rate		1 × 10^−3^
Batch size		32
Number of epochs		200
Data augmentation		Gaussian noise with σ = 0.005, augmentation factor = 10
Anomaly threshold		99th percentile of reconstruction errors on authentic training set
Random seed		42

## Data Availability

The raw hyperspectral datasets generated and analyzed during this study are available from the corresponding author (Yujia Dai, daiyujia@zafu.edu.cn) upon reasonable request due to the large size of the datasets and the specialized nature of the experimental setup. Requests for access will be reviewed and fulfilled in accordance with the journal’s data sharing policies.
